# Low Hemoglobin among Pregnant Women in Midwives Practice of Primary Health Care, Jatinangor, Indonesia: Iron Deficiency Anemia or *β*-Thalassemia Trait?

**DOI:** 10.1155/2017/6935648

**Published:** 2017-05-29

**Authors:** Ari Indra Susanti, Edhyana Sahiratmadja, Gatot Winarno, Adhi Kristianto Sugianli, Herman Susanto, Ramdan Panigoro

**Affiliations:** ^1^Program Study of Midwifery, Division of Maternal and Child Health, Department of Public Health, Faculty of Medicine, Universitas Padjadjaran, Bandung, Indonesia; ^2^Department of Biochemistry and Molecular Biology, Faculty of Medicine, Universitas Padjadjaran, Bandung, Indonesia; ^3^Department of Obstetrics and Gynecology, Faculty of Medicine, Universitas Padjadjaran and Dr. Hasan Sadikin General Hospital, Bandung, Indonesia; ^4^Department of Clinical Pathology, Faculty of Medicine, Universitas Padjadjaran and Dr. Hasan Sadikin General Hospital, Bandung, Indonesia

## Abstract

Low hemoglobin (Hb) or anemia is common among pregnant women in developing countries which may cause adverse pregnancy outcomes and maternal deaths. Our study aimed to assess Hb level measured by midwives in primary health care facility at rural area of Jatinangor, Indonesia, and to explore whether the anemia was due to iron deficiency (IDA) or *β*-thalassemia trait (*β*-TT). Pregnant women (*n* = 105) had finger prick test for Hb level during a regular antenatal care examination from October to November 2016. Hb level by finger prick test was compared with venous blood, measured by complete blood count (CBC). Indices including MCV and MCH and indices of Shine & Lal, Mentzer, Srivastava, Engels & Frase, Ehsani, and Sirdah were analyzed to differentiate anemia due to IDA and anemia due to suspect *β*-TT. HbA2 was measured to confirm *β*-TT. Anemic pregnant women were found in 86.7% by finger prick test compared to 21.9% (*n* = 23) by CBC. The prevalence of *β*-TT in our study was 5.7%. Hb measurement among pregnant women in low resource area is highly important; however, finger prick test in this study showed a high frequency of anemia which may lead to iron oversupplementation. A standard CBC is encouraged; MCV and MCH would help midwives to identify *β*-TT.

## 1. Introduction

Iron deficiency is the leading nutrient deficiency in the world, resulting in iron deficiency anemia (IDA) that affects infants, young children, and women of child-bearing age [1]. In pregnancy, lower hemoglobin occurs as a physiological phenomenon especially in the second trimester. Iron need is increased as a result of higher iron demand to accommodate the requirement of fetal placental unit [2]. Since anemia in pregnancy may lead to adverse pregnancy outcomes and maternal deaths [3], iron supplementation and proper nutrition are needed to fulfill the iron deficiency in pregnancy [4].

Iron deficiency is not the only etiology of anemia in the developing countries. Other coinfections, for example, helminthes, tuberculosis, and HIV, or other diseases such as diabetes mellitus may exist [5]. Interestingly, iron deficiency has a protective role for malarial infection, and iron supplementation in this malarial endemic area may thus increase the infection susceptibility [6]. Therefore, a good control of anemia in low- and middle-income countries needs to be considered [7]. Furthermore, low Hb can be caused by hemoglobinopathies, especially in area where thalassemia trait prevalence is high [8]. Individuals with thalassemia carrier may have enough iron due to erythropoiesis by hepcidin suppression, and excessive iron supplementation may subsequently increase the risk for various complications [9]. Whether the anemia in pregnancy in this area is due to iron deficiency or other causes needs to be further explored.

In areas with limited resources, anemia in pregnancy has been often examined by low cost test, which is an incredibly useful tool, that is, capillary finger-pricked test. On the other hand, in area where thalassemia prevalence is high, thalassemia trait is performed with inexpensive tool that has a good validity test, including Naked Eye Single Tube Red Cell Osmotic Fragility Test (NESTROFT) [10]. NESTROFT is, however, sometimes not possible to be done in the field because it needs some preparation. Other tools that can predict thalassemia trait are venous complete blood count (CBC) indices, which vary from indices using RBC, MCV, and MCH parameters, including Shine & Lal, Mentzer, Srivastava, Engels & Frase, Ehsani, and Sirdah index, to indices using RDW parameter including Green & King index [11]. In the case of pregnant women, HbA2 level is used to predict *β*-thalassemia trait (*β*-TT) [12]. The definitive differential diagnosis between *β*-TT and IDA can be based on the result of HbA2 electrophoresis, serum iron levels, and a ferritin calculation [13].

In Indonesia, the prevalence of anemia among pregnant women is declining over the time, but the number is still as high as more than 35% [14]. Since this country is located in the thalassemia belt, exploring thalassemia carrier in anemic pregnant women especially by midwives in limited source setting becomes an important issue. Midwives play a major role in antenatal care in the primary health care facility in rural area. Iron tablets are often prescribed without proper blood examination but by recognizing clinical symptoms [15]. This study aimed to explore the hemoglobin (Hb) level among pregnant women measured by midwives in the primary health care facility in rural area of West Java Province, Indonesia. The etiology of anemia in pregnancy was also assessed whether the anemia was due to iron deficiency or *β*-TT.

## 2. Materials and Methods

### 2.1. Pregnant Women Inclusion

The study was conducted using cross-sectional analytic comparative design. All pregnant women who visited the primary health care facility at Jatinangor, West Java, between October and November 2016 were invited to take part in this study after consent. In brief, while waiting in the waiting room for a regular antenatal care, the pregnant women and their husbands were given information about anemia including the possibility and the effect of anemia due to IDA and *β*-TT in the pregnancy. Known diseases such as diabetes mellitus and positive HIV were excluded.

### 2.2. Anemia Status Measurement and Hemoglobin Analyses

The anemia status (Hb level < 11 g/dL) [16] of pregnant women was determined from capillary blood from a finger-pricked test (EasyTouch® GCHb) by midwives who had a preeducated finger-pricked technique according to Indonesian Ministry of Health document (MOH, number 43, 2013). After signing the informed consent form, venous blood samples were drawn and stored at 2–8°C before being transported to Dr. Hasan Sadikin General Hospital, Bandung. The distance between the primary health care facility and the hospital was approx. 50 km and the time between the blood draw and measurement was about 4 hours. Automated hematology analyzer with impedance method for complete blood count (CBC) measurement (Sysmex XP-100, Japan) was used to measure 8 parameters including Hb, mean corpuscular volume (MCV), and red blood count (RBC). Various indices were calculated including index of Shine & Lal (MCV^2^*∗*MCH/100), Mentzer (MCV/RBC), Srivastava (MCH/RBC), Engels & Frase (MCV − RBC − (5*∗*Hb)), Ehsani (MCV − 10*∗*RBC), and Sirdah (MCV − RBC − 3*∗*Hb), with cut-off of IDA and suspect *β*-TT as shown in Table 1. To confirm *β*-TT, hemoglobin capillary electrophoresis was performed to measure HbA2 levels (MG300, Sebia, France). Elevated HbA2 level (≥3.5%) was designated as *β*-TT. Study protocol had been reviewed and ethical clearance was granted by Ethical Committee of Faculty of Medicine, Universitas Padjadjaran, number 453/UN6.C1.3.2/KEPK/PN/2016.

### 2.3. Statistical Analyses

Data was collected into paper-based form and inputted into predesigned Excel sheet (Microsoft Corp., USA). The data distribution of CBC result was tested for normality using Shapiro-Wilk test; when the test pointed out that *p* value was >0.05, the mean and standard deviation were calculated and when *p* value was <0.05, the median and min-max value were calculated.

Characteristics of the pregnant women were described and the frequencies of anemia using finger prick test and CBC were tabulated and compared; *p* value was considered significant when *p* < 0.05 (Student's *t*-test). Concordance of Hb level between finger prick test and CBC test and also between various indices and HbA2 analyses were calculated to determine Cohen's kappa value [17]. Analyses were performed on STATA 12.0 (Stata Corp., Texas, USA).

## 3. Results

### 3.1. Anemia Prevalence in Pregnancy

In total, there were 105 pregnant women included in this study with median age of 29 (range: 18–39) years. The finger prick test's result showed that 91 (86.7%) pregnant women had anemia, of whom 71.4% had mild anemia (Hb > 8 g/dL) and 15.2% had moderate anemia (Hb: 6.5–8 g/dL). Interestingly, while using CBC method, mild anemia was found in only 23 (21.9%) women (Table 1). There was, thus, a significant difference in frequency of anemia in pregnant women using finger prick test and CBC, that is, 86.7% and 21.9%, respectively, and this discordance result in both measurements had an agreement level (Cohen's kappa) of 0.056, which was very poor. Since the anemia prevalence using CBC was around the national prevalence of anemia published earlier [14], we used the CBC value for further analyses.

Anemia status in pregnant women was shown according to the gravida and pregnancy trimester (Table 2). There was a correlation between trimester and anemia status; that is, anemia was more prevalent in later stage of pregnancy (*p* = 0.021, Spearman's correlation), while there was no correlation between anemia status and the number of gravidas (*p* = 0.997).

### 3.2. Analyses of Complete Blood Count Indices

Further analyses of anemic pregnant women (*n* = 23) using CBC showed that single parameter of MCV <76 fL and MCH <24 pg and index of Shine & Lal could detect suspect *β*-TT for 5, 6, and 7 pregnant women, respectively, compared to other indices which could detect much lower number (Table 3). A total of 6 pregnant women had HbA2 result of >3.5, of whom 5 had *β*-TT and 1 was suspected as having probably HbE because the HbA2 result was very high. The HbA2 result indicated that the prevalence of *β*-TT in our study among pregnant women was at least 5.7%. Interestingly, when the CBC indices were compared with HbA2 analyses, discordance results were found with Cohen's kappa ranging from 0.20 to 0.25, which had a fair quality of agreement. Since the number of anemic pregnant women was low (*n* = 23), we did not calculate the sensitivity and specificity between both CBC indices and HbA2 analyses.

## 4. Discussions

During a regular antenatal care in the primary health care facility in a rural area in Jatinangor, Indonesia, Hb level is measured to control the well-being of both mother and baby. Our study showed that anemia in pregnancy status measured by midwives using inexpensive and easy to perform capillary finger prick test was very high, reaching almost 90% of pregnant women. Capillary finger prick test is a simple test to perform; nevertheless, this test requires proper technique. Therefore, the use of finger prick test by midwives in primary health care facility needs to be further reviewed.

When the Hb level is measured using CBC, result showed that anemia among pregnant women in our study only occurred in around 20%, in line with the national prevalence of anemia published earlier [14]. The discrepancy result shown using finger prick test and standard CBC seems too high and may lead to overprescription of iron supplementation. Iron is indeed needed more in second and third semesters as a result of increased fetal placental unit [18]. This conforms to our study, showing that anemia was more prevalent in third semester (*p* = 0.021). Iron deficiency anemia in later phase of pregnancy is associated with the birth weight of the neonates [19] and may play a role as a major killer in pregnant women [7]. Appropriate iron supplementation is needed in pregnancy and WHO has guidelines for iron supplementation in pregnant women and individuals where infection and hemoglobinopathies prevalence are high [1].

The first blood taken in life among healthy individual happens particularly in pregnant women. Study in the neighboring country Thailand showed that the main causes of anemia among pregnant women are IDA and thalassemia carriers [20]. In Indonesia, Thalassemia ranks 6th for catastrophic diseases; therefore, there is a rising interest to screen thalassemia carrier in the general population. Our study has shown *β*-TT prevalence as high as 5.7%, conforming to the national prevalence which is around 6 to 10%. Although the national guidelines for thalassemia trait screening exist [21], thalassemia trait screening in general population is not regularly conducted yet. Moreover, the knowledge about etiology of anemia among pregnant women other than iron deficiency such as hemoglobinopathies is not well known among students midwifery (Sahiratmadja et al., unpublished data), making the question on how to distinguish IDA and thalassemia trait a major concern, especially in the limited resource area. IDA can be diagnosed by simple and easy detection. Red blood cells are smeared in object glass and could be seen as small or microcytic cells with hypochromic color. More accurately, IDA can be diagnosed by calculating the CBC performed by a hematology analyzer. Furthermore, the *β*-TT pregnant women are often misdiagnosed as those suffering from iron deficiency anaemia; thus, they are given unnecessary iron medication. In this setting, a reliable screening test becomes a need of the hour for screening purposes.

In meta-analysis study to distinguish individuals with *β*-TT and IDA, Hoffmann et al. (2015) have noted that microscopic smear examinations and CBC are difficult to be performed in rural area since the source and personnel to examine the smear are scarce, and CBC analyzer is expensive and not portable [22]. For example, in rural area where equipment or expertise is scarce, One Tube Osmotic Fragility Test (OTOFT) is a simple test that can be done to examine carriers [23]. Interestingly, another study also showed that HbA2 is reliable to measure *β*-TT when IDA also coexists [24]. Ideally, HbA2 is more reliable as indicated to detect *β*-TT cases in pregnant women [12], but this measurement is costly. One obstacle in HbA2 measurement is that *α*-thalassemia could be coinherited with *β*-thalassemia, making HbA2 level have normal value, leading to difficulty in screening *α*-thalassemia using HbA2 level [25]. To confirm the definitive diagnosis, DNA analysis is needed.

Further study needs to be designed to calculate the cost-effectiveness screening in primary care [26], that is, the strategy of timing of screening of thalassemia carrier. Whether the screening is more beneficial during premarital screening [27, 28] or in young adults [29] needs further exploration. Moreover, knowledge of etiology of anemia among student midwives needs to be strengthened for thalassemia problem. A simple standard CBC, that is, low MCV and low MCH, may be of important role to detect carriers and a good training program for the midwives leading the process is urgently required.

Limitation of this study was that iron status was not assessed, as this study was not designed for iron status study, particularly since this study was done in a limited setting area. Other limitation is that low number of anemic pregnant women in our study is considered low; however, the consistent results of the currently used method for Hb, that is, prick test, showed no doubt that the finger prick method has poor reliability.

## 5. Conclusion

Low hemoglobin level by finger prick test measured by midwives showed much higher prevalence of anemia, leading to iron oversupplementation. Since Indonesia is located in thalassemia belt area, a reliable measurement to distinguish anemia of iron deficiency or thalassemia carrier is highly needed. Simple single parameter of CBC such as MCV and MCH would help midwives to identify *β*-TT among pregnant women; therefore, wherever it is possible, a standard CBC to detect *β*-TT is encouraged.

## Figures and Tables

**Table 1:** Characteristics and distribution of anemia among pregnant women (*n* = 105) in Jatinangor Puskesmas, West Java, Indonesia, using finger prick and CBC methods.

	Finger prick	CBC
Hb value (g/dL); mean (SD)	9.5 (1.4)	11.7 (1.1)
Anemia status; *n* (%)	91 (86.7%)	23 (21.9%)
Mild (Hb: 8–11 g/dL)	75	23
Moderate (Hb: 6.5–8 g/dL)	16	—

*Note*. CBC, complete blood count; Hb, hemoglobin; SD, standard deviation.

**Table 2:** Anemia status among pregnant women (*n* = 105) in Jatinangor Puskesmas, West Java, Indonesia, stratified by gravidity and trimester of pregnancy.

	Anemia *N* = 23	Not anemia *N* = 82	*p* value
Gravidity			
Primigravida	7	23	
Multigravida (2-3)	12	47	
Grand multigravida (≥4)	4	12	0.997
Trimester			
(1) (<12 weeks)	—	10	
(2) (12–26 weeks)	7	35	0.021^*∗*^
(3) (>26 weeks)	16	37	

*Note*. ^*∗*^*p* value was set as <0.05 (Spearman's correlation).

**Table 3:** Concordant result of CBC indices to HbA2 electrophoresis among pregnant women (*n* = 23) in Jatinangor Puskesmas, West Java, Indonesia.

Indices	HbA2 < 3.5 (*n* 17)	HbA2 ≥ 3.5 (*n* 6)	Total	*K*
RBC count				
IDA < 5	17	5	22	0.228
Susp. *β*-TT > 5	—	1	1	
MCV^*∗*^				
IDA >76	15	3	18	0.232
Susp. *β*-TT < 76	2	3	**5**	
MCH^*∗*^				
IDA > 24	14	3	17	0.203
Susp. *β*-TT < 24	3	3	**6**	
Shine & Lal^*∗*^				
IDA > 1530	13	3	16	0.251
Susp. *β*-TT < 1530	4	3	**7**	
Mentzer				
IDA > 13	17	5	22	0.228
Susp. *β*-TT < 13	—	1	1	
Srivastava				
IDA > 38	17	5	22	0.228
Susp. *β*-TT < 38	—	1	1	
Ehsani				
IDA > 15	17	5	22	0.228
Susp. *β*-TT < 15	—	1	1	
Sirdah				
IDA > 27	17	5	22	0.228
Susp. *β*-TT < 27	—	1	1	
Engels Fraser				
IDA < 0	17	6	23	n.d.
Susp. *β*-TT > 0	—	—	—	

*Note*. Susp. *β*-TT, suspect *β*-thalassemia trait; IDA, iron deficiency anemia; *K*, Cohen's kappa. ^*∗*^The indices with high suspect *β*-thalassemia trait cases.

Formula to calculate indices as shown in the bracket: index of Shine & Lal (MCV^2^*∗*MCH/100), Mentzer (MCV/RBC), Srivastava (MCH/RBC), Engels Frase (MCV − RBC − (5*∗*Hb)), Ehsani (MCV − 10*∗*RBC), Sirdah (MCV − RBC − 3*∗*Hb).
